# Seroprevalence of chlamydial infection in dairy cattle in Guangzhou, southern China

**DOI:** 10.1186/2046-0481-66-2

**Published:** 2013-02-05

**Authors:** Dong-Hui Zhou, Fu-Rong Zhao, Hui-Yan Xia, Min-Jun Xu, Si-Yang Huang, Hui-Qun Song, Xing-Quan Zhu

**Affiliations:** 1 State Key Laboratory of Veterinary Etiological Biology, Lanzhou Veterinary Research Institute, Chinese Academy of Agricultural Sciences, Lanzhou, Gansu Province, 730046, People’s Republic of China; 2 College of Animal Science and Technology, Yunnan Agricultural University, Kunming, Yunnan Province, 650201, People’s Republic of China

**Keywords:** Chlamydia, Dairy cattle, Seroprevalence, Indirect hemagglutination antibody (IHA), Guangzhou, China

## Abstract

*Chlamydia* spp. are obligate intracellular gram-negative bacteria that cause a wide range of significant diseases in humans and animals worldwide, resulting in significant economic losses. Chlamydial infection in cattle has been reported in many countries including China. However, there has been no survey of chlamydial infection of dairy cattle in Guangzhou, southern China. The objective of the present investigation was to examine the chlamydial seroprevalence in dairy cattle in Guangzhou, subtropical southern China by using an indirect hemagglutination assay (IHA). The overall seroprevalence of chlamydial infection in dairy cattle was 7.25% (29/400). Greater than or equal to eight-yr-old dairy cattle had the highest seroprevalence (10.34%), followed by those that were ≥ 6 years old or < 7 years old dairy cattle (10.20%), although there were no statistically significant differences among different groups (*P* > 0.05). Dairy cattle with 5 pregnancies had the highest seroprevalence (10.81%). These results indicate that chlamydial infection was present in dairy cattle in Guangzhou, subtropical southern China, and integrated strategies and measures should be executed to control and prevent chlamydial infection and disease outbreak in the study region.

## Background

*Chlamydia* spp. are obligate intracellular gram-negative bacterial pathogens that cause a wide range of significant diseases with huge economic losses in humans, birds and other animals worldwide [1-5]. Chlamydiaceae only has a single genus *Chlamydia* that comprises nine species according to the new taxonomy in 2011 in the new edition of Bergey’s Manual of Systematic Bacteriology [6]. Chlamydial infections in cattle have been described worldwide and cause disease syndromes such as pneumonia, enteritis, conjunctivitis, polyarthritis, encephalomyelitis, mastitis, arthritis, infertility, abortion and other urogenital tract infections (endometritis, repeat breeding, vaginitis, seminal vesiculitis) as well as subclinical infections [1,2,5]. Several chlamydial species infecting cattle are occasionally transmitted to humans when humans are exposed to the birth fluids and placentas of infected animals [2].

Chlamydial infection in cattle has been reported in many countries such as Australia, Germany, Ireland, Italian, Sweden, Switzerland [7-12], as well as China. In the present paper, Table 1 summarizes surveys of chlamydial infection in cattle in some provinces of the People’s Republic of China (PRC) which were published in the Chinese language in local journals and are not readily accessible to international readers. However, there has been no reported survey of chlamydial infection in dairy cattle in Guangzhou, southern China. The objective of the present investigation was to examine the seroprevalence of chlamydial infection in dairy cattle in Guangzhou, the capital of Guangdong province, southern China. The results may provide base-line data for the implementation of integrated strategies to prevent and control of chlamydial infection in dairy cattle in this region.

**Table 1:** Prevalence of chlamydial infection in cattle in People’s Republic of China (PRC) examined by indirect hemagglutination test (IHA)

**Region**	**No. tested**	**Prevalence (%)**	**Time tested (year)**	**Reference**
Gansu	124	17.7	1997-1998	[18]
Ningxia	380	28.4	2007	[16]
Hebei	13	23.1	1997-1998	[18]
Henan	35	25.7	1997-1998	[18]
Qinghai	321	5.90	2009	[17]
Shandong	62	16.1	1997-1998	[18]
Shanxi	132	43.2	1997-1998	[18]
Sichuan	39	25.6	1997-1998	[18]

## Methods

### The study site

The survey was conducted in Guangzhou City which is the capital of Guangdong Province, China and has a subtropical monsoon climate. Crossed by the Tropic of Cancer, it is located between longitude 112°57' to 114°3' east and latitude 22°26' to 23°56' north, bordering on the South China Sea. It is the biggest cosmopolitan city in South China, and also China's Southern Gateway to the world. The annual average temperature is 22.8°C, the average relative humidity is about 68%, and the annual rainfall at the urban area is over 1600 mm. The city covers a total area of 7434.4 square kilometers and has a population of approximately twelve millions.

### Collection of serum samples

Blood samples were collected randomly from 400 dairy cattle (representing different ages and pregnancies) on 5 representative farms between July 2009 and March 2010 in Guangzhou City, Guangdong Province, China. The dairy cattle populations represented a local breed (Chinese Holstein) and introduced breed (American/Australian Holstein-Friesian and British Jersey). The animals of each herd were randomly selected, and 1 blood sample was collected from each animal. Approximately 5 ml of blood were obtained via a jugular vein. Blood samples were centrifuged at 3,000 rpm for 10 min, and serum was obtained, frozen, and stored at −20°C until further analysis. Biometric data for dairy cattle, including ages, breeds and numbers of past pregnancies were obtained through a questionnaire at the time of blood collection.

### Serological examination

Antibodies to *Chlamydia* were determined in sera using an indirect hemagglutination assay (IHA) with a commercially available kit (Lanzhou Veterinary Research Institute, Chinese Academy of Agricultural Sciences, Lanzhou, Gansu Province, China) according to the manufacturer's instructions as described previously [13,14]. IHA is a sensitive and specific technique for measuring mixed chlamydial antibodies (IgG and IgM) [15], which has been used extensively in several animals in China [13,14,16]. In brief, 75 μL of IHA dilution solution was transferred into a 96-well V bottomed reaction plate with 25 μL of serum sample added and mixed gently with pipette. About 25 μL of the mixture was 4-fold gradually diluted into another 2 holes and 25 μL mixture in the third hole was discarded at last to maintain 75 μL system. The dilution in the 3 wells was 1:4, 1:16 and 1:64, respectively. Positive, negative and blank controls were included at the same plate. After 25 μL *Chlamydia* antigen was added to each well, the plate was shaken slightly with a vibrator for 2 min followed by incubation at 37°C for 2 h. The test was considered positive when a layer of agglutinated erythrocytes was formed in wells at dilutions of 1:16 or higher, and positive and negative controls were included in each test.

### Statistical analysis

Differences in seroprevalence of chlamydial infection among dairy cattle of different age groups and numbers of pregnancies were analyzed using a Chi square test using the SPSS for Windows (Release 18.0 standard version, SPSS Inc., Chicago, Illinois). The differences were considered statistically significant when *P* < 0.05.

## Results

A total of 400 serum samples from dairy cattle in Guangzhou, Southern China were examined by IHA for chlamydial antibodies. 29 of 400 (7.25%) examined dairy cattle were seropositive for chlamydial infection by IHA (Table 2). Different levels of seropositivity were detected among the 5 different farms, namely 6.25%, 8.0%, 8.33%, 7.5% and 6.67% of the examined samples from farms A, B, C, D and E were chlamydial antibody-positive, respectively (Table 2).

**Table 2:** Seroprevalence of chlamydial infection in dairy cattle in Guangzhou, southern China examined by indirect hemagglutination assay (IHA)

**No. farm**	**No. examined**	**No. positive**	**Prevalence (%)**
A	80	5	6.25
B	75	6	8.0
C	60	5	8.33
D	80	6	7.5
E	105	7	6.67
Total	400	29	7.25

The ages of the examined dairy cattle ranged between 1 year and 8 years, seroprevalence varied in different age groups, ranging from 1.96% to 10.3% (Table 3). The numbers of parturition of dairy cattle ranged between 0 and 7 pregnancies, seroprevalence varied in dairy cattle with different numbers of pregnancies, ranging from 4.54% to 10.8% (Table 4). The highest seroprevalence was found in dairy cattle with five pregnancies (10.8%), followed by dairy cattle having 3 pregnancies (10%), although there were no statistically significant differences among different groups (*P* > 0.05).

**Table 3:** Seroprevalence of chlamydial infection in dairy cattle of different ages in Guangzhou, southern China examined by indirect hemagglutination assay (IHA)

**Age (year)**	**No. examined**	**No. positive**	**Prevalence (%)**
1 ≤ yr < 2	44	2	4.55
2 ≤ yr < 3	51	1	1.96
3 ≤ yr < 4	54	4	7.41
4 ≤ yr < 5	25	1	4.0
5 ≤ yr < 6	91	8	8.79
6 ≤ yr < 7	49	5	10.20
7 ≤ yr < 8	57	5	8.77
≥ 8	29	3	10.34
Total	400	29	7.25

**Table 4:** Seroprevalence of chlamydial infection in dairy cattle with different numbers of pregnancies in Guangzhou, southern China examined by indirect hemagglutination assay (IHA)

**No. pregnancy**	**No. examined**	**No. positive**	**Prevalence (%)**
0	60	3	5.0
1	72	6	8.33
2	64	4	6.25
3	40	4	10.0
4	66	5	7.58
5	37	4	10.81
6	44	2	4.55
≥ 7	17	1	5.88
Total	400	29	7.25

## Discussion

*Chlamydia* can infect epithelial cells and monocyte or macrophages of a wide host range. Chlamydial infection in cattle has been reported sporadically all over the world and is implicated in respiratory and reproductive tract diseases [2,5]. Though direct evidence of the infectious agent is the ultimate diagnosis, sero-assays are more suitable for screening large numbers of samples. The complement-fixation test (CFT) is the most-widely used and accepted serological test for diagnosing chlamydiosis. However, an indirect hemagglutination assay (IHA) is described for detecting chlamydial antibodies in psittacosis diagnostic sera. The Microtiter-IHA test was more sensitive than the CFT [15].

In the present survey, chlamydial antibodies were detected in 29 (7.25%) of 400 dairy cattle by IHA (Table 2). The overall seroprevalence was lower than that reported in Gansu, Hebei, Henan, Ningxia, Shandong, Shanxi and Sichuan in China (Table 1), and some other countries or regions such as Germany, Italian and Switzerland [7,9,10], but higher than that documented in Qinghai [17], Sweden and Ireland [11,12]. These differences may due to different diagnostic methods used, cattle of different sources and surveyed, and samples from different regions.

As shown in Table 3, the seroprevalence varied in different age groups (1.96% to 10.34%), with dairy cattle of greater than or equal to eight-yr-old having the highest seroprevalence of 10.34%, followed by dairy cattle of ≥ 6 years old or < 7 years old (9.61%). However, there were no statistically significant differences between different age groups (*P* > 0.05). The varied seroprevalence in different age groups suggests the possibility of horizontal transmission in the investigated herds. The highest chlamydial seroprevalence in dairy cattle of greater than or equal to eight-yr-old may due to chronic infection or sustain infection, also may due to lower resistance or immunity of the dairy cattle of this age. The association between chlamydial seroprevalence and numbers of pregnancies was also analyzed in the present study (Table 4), and varied chlamydial seroprevalence was detected. The seroprevalence in dairy cattle with five pregnancies was the highest (10.8%), followed by dairy cattle with 3 pregnancies, but the differences were not statistically significant (*P >* 0.05).

## Conclusion

The results of the present survey indicated that chlamydial infection is prevalent in dairy cattle in Guangzhou, subtropical southern China, which may represents one of the causes of bovine abortion, pneumonia and arthritis. Therefore, integrated strategies and measures should be performed to control and prevent chlamydial infection and disease outbreak in dairy cattle in the study region.

## Abbreviations

PRC: People’s Republic of China; IHA: Indirect hemagglutination assay; CFT: Complement-fixation test.

## Competing interests

The authors declare that they have no competing interests.

## Authors’ contributions

DHZ conceived and designed the study, and critically revised the manuscript. FRZ, HYX and MJX performed the experiments, analysed the data and drafted the manuscript. SYH, HQS and XQZ helped in study design, study implementation and manuscript revision. All authors read and approved the final manuscript.
